# Plasticity in the growth of body segments in relation to height‐for‐age and maternal education in Guatemala

**DOI:** 10.1002/ajhb.23376

**Published:** 2019-12-19

**Authors:** Luis Ríos, José Manuel Terán, Carlos Varea, Barry Bogin

**Affiliations:** ^1^ Department of Physical Anthropology Aranzadi Science Society San Sebastian Spain; ^2^ Department of Biology, Faculty of Sciences Universidad Autónoma de Madrid Madrid Spain; ^3^ School of Sport, Exercise & Health Sciences Loughborough University Loughborough UK

## Abstract

**Objectives:**

Plasticity in the growth of body segments between populations has been researched in relation to migration, temporal change and high‐altitude studies. We study the within population variation in body segments, thus controlling for some of the environmental and genetic differences that could be at play in between populations studies. We test a version of the thrifty phenotype hypothesis, where the growth of head‐trunk and hand are prioritized due to their functional significance over height and leg growth.

**Materials and methods:**

A total of 3913 Guatemalan, rural, semi‐urban and urban, Maya and Ladino children 6 to 15 years old were studied. Height, sitting height, leg length, and metacarpal length were studied in relation to three proxies for living conditions: height‐ and leg length‐for‐age, and maternal education. Estimation statistics and null hypothesis significance testing were used to analyze the data.

**Results:**

Metatarsal length and sitting height values were higher than height and leg length respectively. Relative metacarpal length was conserved across height‐for‐age groups. Females were less affected than males for metacarpal length and sitting height, but more affected for leg length.

**Conclusion:**

Our results agree with the thrifty phenotype hypothesis, where metacarpal and sitting height growth would be prioritized over height and leg length due to greater functional significance.

## 1 INTRODUCTION

Plasticity refers to the ability of an organism to modify its biology to respond to changes in the environment, particularly when these changes are physiologically stressful (Bogin, 1999). Research on plasticity in growth has analyzed the change in the lengths of body segments in samples living in different environments (Bogin, Smith, Orden, Silva, & Loucky, 2002; Padez, Varela‐Silva, & Bogin, 2009; Payne, Kumar, Pomeroy, Macintosh, & Stock, 2018; Pomeroy et al., 2012), or belonging to different generations (Tanner, Hayashi, Preece, & Cameron, 1982). In these studies it has been shown that, in conditions of environmental stress, leg length is often more sensitive than is the head‐trunk segment (Bogin et al., 2002; Bogin & Varela‐Silva, 2009; Bogin & Varela‐Silva, 2010; Leitch, 1951; Tanner et al., 1982), and that tibia and ulna lengths are also more sensitive than humerus, hand and foot lengths (Payne, Kumar, et al., 2018; Pomeroy et al., 2012). A proximate reason for this sensitivity is due to the allometry of skeletal growth, where the more proximal body segments grow fastest prenatally and are less exposed to extra‐uterine environmental stress, but more distal segments grow most rapidly after birth and are more exposed to environmental stress (Bogin & Varela‐Silva, 2010).The thrifty phenotype hypothesis (Hales & Barker, 1992; Wells, 2013) offers an ultimate evolutionary explanation; the growth of human body segments with greater functional significance, such as “head‐brain,” “trunk‐major organs,” and “hands‐tools” will be prioritized over forearms and legs.

The differential growth of hand, head‐trunk and other body segments have been recently studied in populations living in places associated to diverse environmental conditions (Payne, Kumar, et al., 2018; Pomeroy et al., 2012). In this article, we study the change in metacarpal length and sitting height associated with three measures of overall living conditions widely used by epidemiologists, public health researchers, economists, and anthropologists: height‐ and leg length‐for‐age and maternal education. We do this within the same samples of children and, thus, can exclude any strong influence of possible genetic or environmental differences associated to groups living in different places or periods.

## 2 MATERIALS AND METHODS

Two samples of Guatemalan children from 6 to 15 years of age were studied, the Universidad del Valle de Guatemala sample (UVG, 1777 children), and the Universidad de San Carlos de Guatemala sample (USAC, 2136 children). The UVG sample belongs to the UVG Longitudinal Study of Child and Adolescent Development, initiated in 1953 and whose history and protocols have been reviewed in detail elsewhere (Bogin, 2018; Nikitovic & Bogin, 2014). The UVG Maya sample was formed by children attending two national schools with no fee located in San Pedro Sacatepequez, 24 km northwest of Guatemala City. These children represent a low socioeconomic status (SES) sample (Bogin & Macvean, 1984), and measurements were recorded between 1979 and 1999. The UVG Ladino sample was formed by children from Guatemala City attending a national school with no fee, representative of the low SES population of Guatemala City (Bogin & Macvean, 1983), and measurements were recorded between 1965 and 1999, mostly between 1970 and 1985. The USAC sample was formed by two main groups of children: low SES, mostly Maya, children from rural villages from six municipalities (Comalapa, Cuilco, Retalhuleu, San Bartolomé Milpas Altas, San Juan La Laguna, Sololá), and high SES Ladino children from six private schools from Guatemala City (Barrantes et al., 1997). Children from rural villages were measured in 1998 and 1999 by a team of anthropologists including present author LR, while children from Guatemala City were measured in 1995 and 1996. As a general SES indicator, for the rural sample, the percentage of children whose mothers did not attend school was 67%, while for the Guatemala City sample, all mothers had at least secondary school education and 45.5% of the mothers had university degrees (Barrantes & Domínguez, 1998). For both studies (UVG and USAC), the common criteria of maternal language and traditional dress, as well as the surname (Spanish or Maya) were used for the distinction between Maya and Ladino (Nikitovic & Bogin, 2014), although this dichotomy, the ascription to each category, and their meanings are the results of complex social and political processes in Colonial and recent Guatemalan history (Bastos, 2007; Bastos & Camus, 2003; Taracena, Gellert, Gordillo, & Sagastume, 2002). A map with the location of the municipal seats from the UVG and USAC samples, with latitude, longitude and altitude data, is shown in Supporting Information 1.

Since the UVG study was semilongitudinal, we only considered children with one measurement and, for those children with more than one observation, we selected randomly only one measurement. For the UVG children, height (H) and metacarpal length (ML) were available. The variable ML was obtained from digitized hand X‐rays with BoneXpert, a programme for automatic analysis of hand X‐rays and bone age (Martin et al., 2011), and consists of the average length of metacarpals 2‐4. Relative segment length for ML was calculated as the absolute ML divided by H (Payne, Kumar, et al., 2018). No SES variables other than school of attendance were recorded for the UVG children. School of attendance was previously shown to be a reliable proxy for parental education and occupation (Bogin & Macvean, 1983). For the USAC children, sitting height (SH) and subischial leg length (LL, obtained by subtracting sitting height from H) were measured. Education of the mother was recorded in the USAC rural Maya sample, and we developed a three‐category scale for this variable (no education, primary first to third grade, primary fourth to sixth grade) to group the children. As in previous research with children (Pomeroy et al., 2012), we used *z* scores in order to adjust the data for age effects and then be able to compare the variables across a range of ages (6‐15 years of age in our case). Since BoneXpert was based on the First Zurich Longitudinal Study (Martin et al., 2011), we used data from this study to obtain *z* scores for ML (Martin et al., 2011) and for H, LL, and SH (Prader, Largo, Molinari, & Issler, 1989), in order to use only one reference sample. The objective was to compare the change in ML (UVG samples) and SH (USAC samples) across the range of variation of H and LL respectively (ML vs H, SH vs LL). Thus, the UVG samples were grouped in four *z*‐score categories of height (<−3, −3/−2, −2/−1, >−1), while the USAC rural Maya and urban Ladino samples were grouped respectively in four (< −3, −3/−2, −2/−1, > −1) or five (−3/−2, −2/−1, −1/0, 0/1, >1) *z*‐score categories of leg length. Descriptive *z*‐score statistics by sample, sex, age, and measurement are presented in the Data S1.

**Figure 1:**
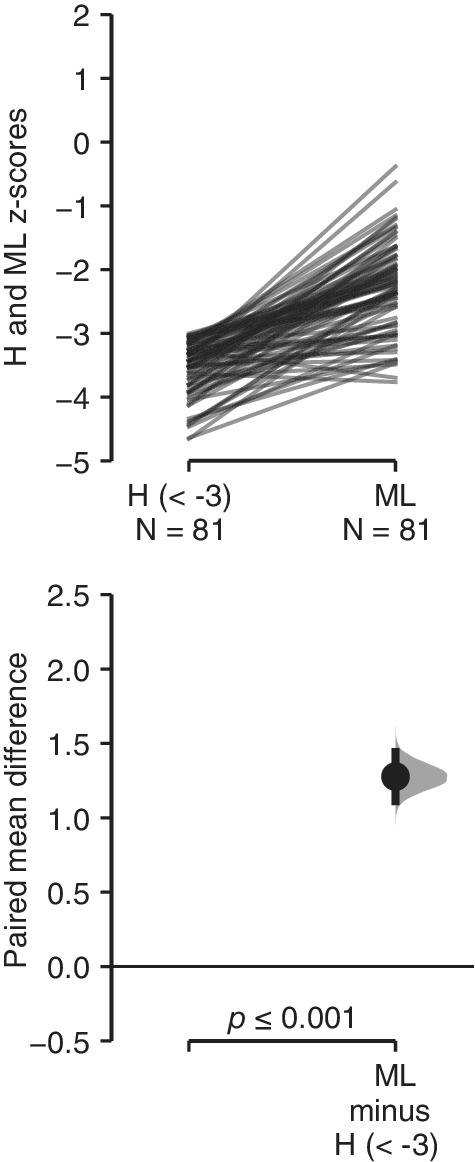
Sample figure illustrating the Tufte slopegraph approach, using a multipaired samples estimation plot to display effect sizes. The figure is composed of two plots. In the upper plot, raw data are presented. Each line represents the *z*‐score values of a paired set of observations for the same child, in this case height (H) and metatarsal length (ML) *z* scores. Sample sizes are indicated for each variable under its abbreviation, in this case N = 81. These are the 87 UVG semi‐urban Maya girls whose *z* score of height (H) was under −3, as indicated by H(<−3). On the lower plot, the paired mean difference is plotted as a bootstrap sampling distribution. The mean difference value between both variables (ML − H(<−3)), depicted as a dot, and the 95% confidence interval, indicated by the ends of the vertical error bars, are shown. The *P* values of the paired *t* test between both variables is shown on the lower axis, in this case *P* ≤ .001

For the sake of clarity, we use estimation statistics for graphic representation (Ho, Tumkaya, Aryal, Choi, & Claridge‐Chang, 2018) (https://www.estimationstats.com/#/), which allows to show the pattern of change in the relation between the variables analyzed, beyond the dichotomous results of the *P* values. The Tufte slopegraph was used as a multi paired samples estimation plot to display effect sizes (ML vs H across *z*‐score categories of H, SH, vs LL across *z*‐score categories of LL). A sample plot is shown and explained in detail in Figure 1. We also include null hypothesis significance testing results. The *P* values from the paired *t* test (ML vs H, SH, vs LL), were calculated with SPSS (Version 20) and included in the figures. To study the change of SH and LL across categories of maternal education, and the change of relative ML length across H categories, the ANOVA test (Tukey post hoc tests) was used. The independent samples *t* test was used to study sexual differences.

## 3 RESULTS

**Figure 2:**
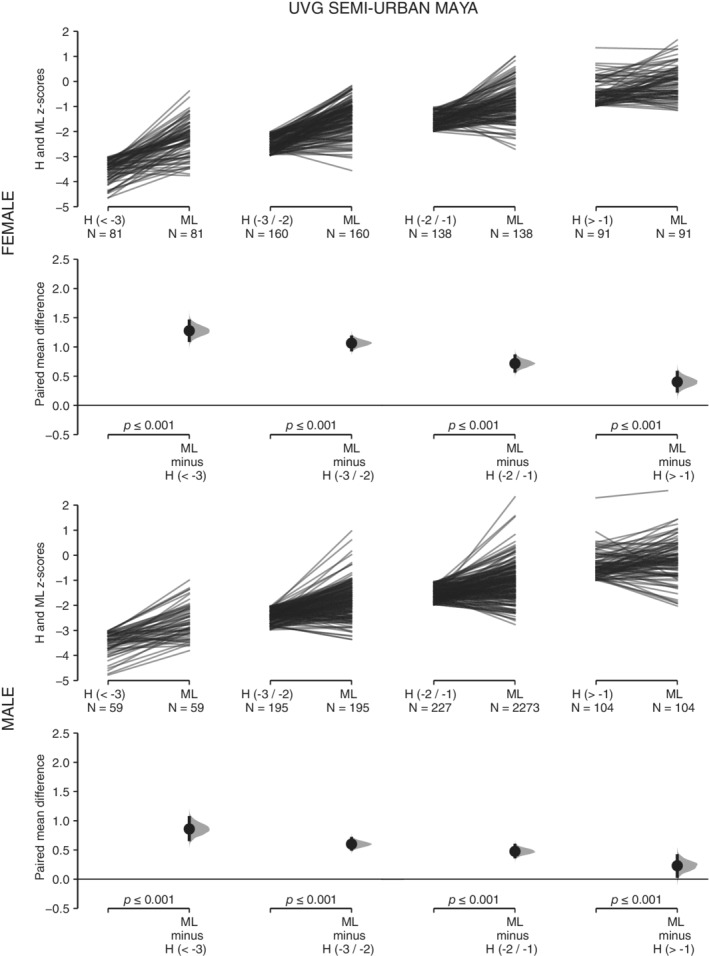
ML vs H in the UVG semi‐urban Maya sample. The comparison of *z* scores between metatarsal length (ML) and height (H) across four categories of H(<−3, −3/−2, −2/−1, >−1) is shown for UVG semi‐urban Maya females (top row) and males (bottom row). See Figure 1 for a description of the details of the plots. The *P* values of the paired *t* test between ML and H values for each H category is shown on the lower axis

**Figure 3:**
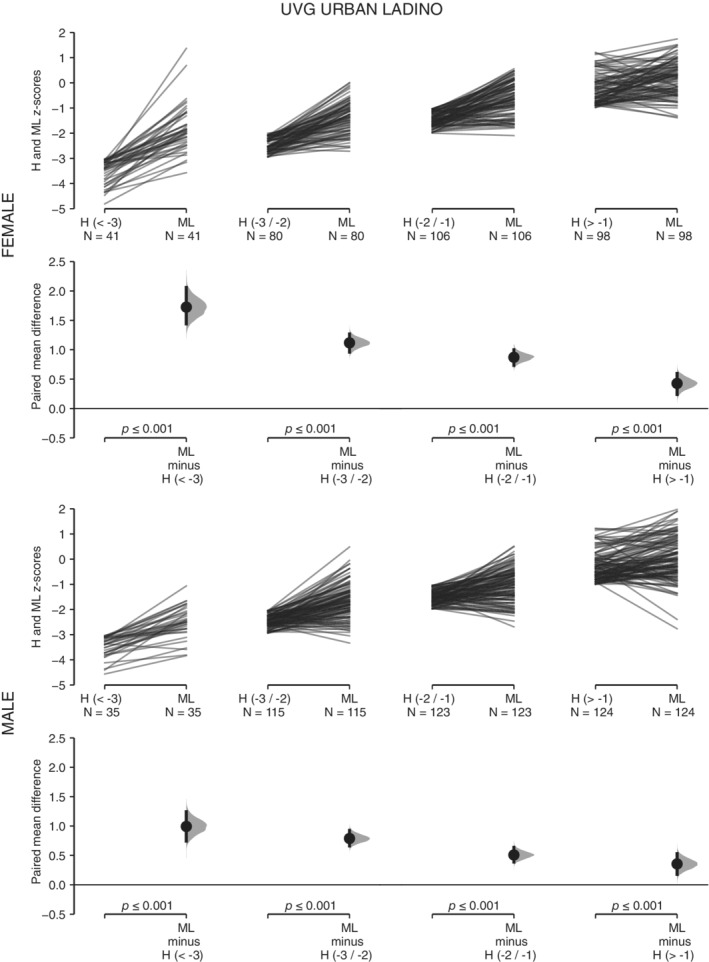
ML vs H in the UVG urban Ladino sample. The comparison of *z* scores between metatarsal length (ML) and height (H) across four categories of H(<−3, −3/−2, −2/−1, >−1) is shown for UVG urban ladino females (top row) and males (bottom row). See Figure 1 for a description of the details of the plots. The *P* values of the paired *t* test between ML and H values for each H category is shown on the lower axis

**Figure 4:**
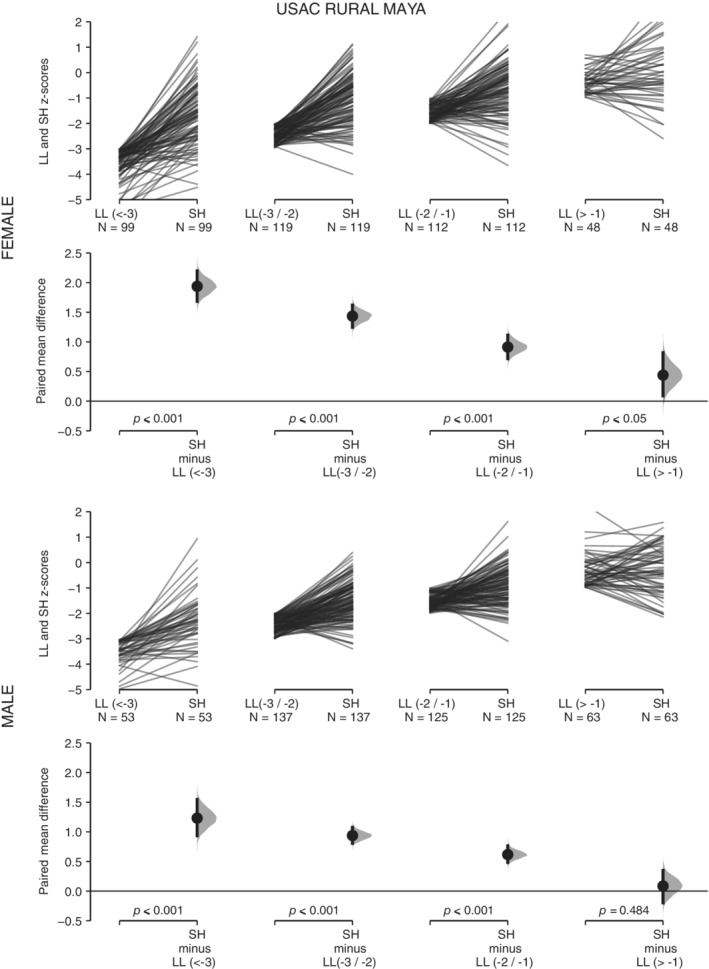
SH vs LL in the USAC rural Maya sample. The comparison of *z* scores between sitting height (SH) and leg length (LL) across four categories of LL (<−3, −3/−2, −2/−1, >−1) is shown for the USAC rural Maya females (top row) and males (bottom row). See Figure 1 for a description of the details of the plots. The *P* values of the paired *t* test between SH and LL values for each LL category is shown on the lower axis

**Figure 5:**
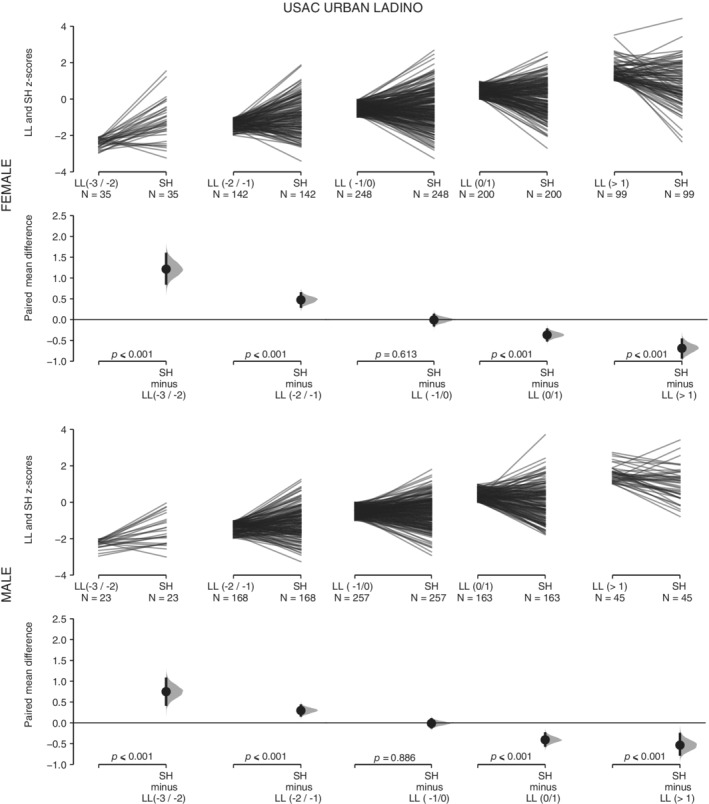
SH vs LL in the USAC urban Ladino sample. The comparison of *z* scores between sitting height (SH) and leg length (LL) across five categories of LL (−3/−2, −2/−1, −1/0, 0/1, >1) is shown for the USAC urban Ladino females (top row) and males (bottom row). See Figure 1 for a description of the details of the plots. The *P* values of the paired *t* test between SH and LL values for each LL category is shown on the lower axis

**Figure 6:**
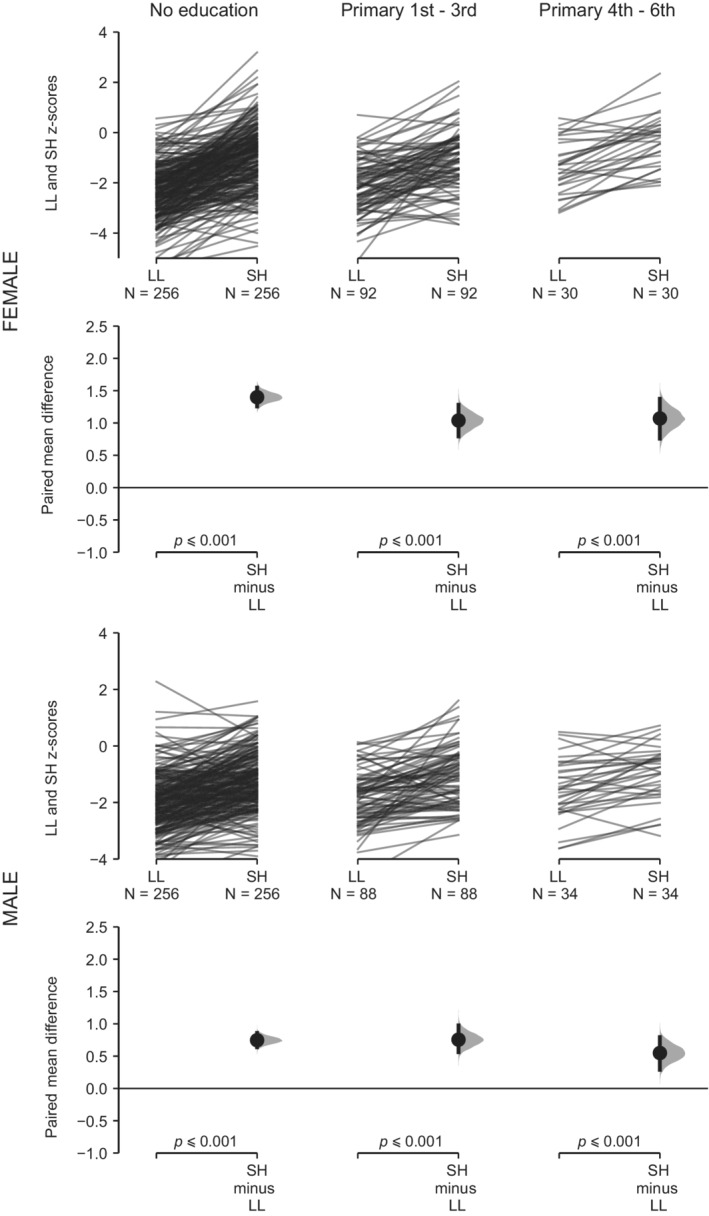
SH vs LL across categories of maternal education in the USAC rural Maya sample. The comparison of *z* scores between sitting height (SH) and leg length (LL) across the three categories of education of the mother is shown for the USAC rural Maya females (top row) and males (bottom row). See Figure 1 for a description of the details of the plots

For the four subsamples of UVG children, ML presents higher *z*‐score values than H across the range of variation of H (Table 1, Figures 2 and 3). These differences are statistically significant in all cases. As H decreases, the same pattern of increase in the mean difference between both variables is observed in the four subsamples (Figures 2 and 3). For the four subsamples of USAC children, SH presents higher *z*‐score values than LL when LL is under −1 *z* score (Table 1, Figures 4 and 5). These differences are statistically significant. Under −1 *z* score of LL, the same pattern of increase in the mean difference between both variables is observed in the four subsamples (Figures 4 and 5). For the high SES USAC urban Ladino samples, this pattern is reversed as LL increases from values higher than −1 *z* score, with higher values for LL than for SH (Figure 5). When we use education of the mother as the grouping variable for the USAC rural Maya children, SH presents higher *z*‐score values than LL in the three categories (Table 2 and Figure 6). These differences are statistically significant in all cases. These results should be considered with caution due to the small sample size for the highest category of maternal education. For the UVG samples, a statistically significant difference for the relative length of ML between categories of H was only observed in the lowest H category for semi‐urban Maya females (Table 3). The differences between sexes are summarized in Tables 4 and 5. Under −1 *z* score of H and LL, higher female ML and SH values were observed (Table 4). These differences were statistically significant except for USAC urban Ladino children. Across categories of education of the mother, a statistically significant difference towards higher female SH and higher male LL was observed in the lowest category (Table 5).

**Table 1:** Mean difference between length of body segments by sample and sex

	Mean difference (ML − H)					
Categories of H	H(<−3)	H(−3/−2)	H(−2/−1)	H(>−1)	H(0/1)	H(>1)
UVG						
Semi‐urban Maya female	1.261***	1.068***	0.716***	0.401***		
Semi‐urban Maya male	0.898***	0.619***	0.484***	0.239***		
Urban Ladino female	1.711***	1.115***	0.870***	0.422***		
Urban Ladino male	0.992***	0.778***	0.503***	0.360***		

	Mean difference (SH − LL)					
Categories of LL	LL(<−3)	LL(−3/−2)	LL(−2/−1)	LL(>−1)	LL(0/1)	LL(>1)
USAC						
Rural Maya female	1.937***	1.434***	0.913***	0.439*		
Rural Maya male	1.230***	0.937***	0.616***	−0.085		
Urban Ladino female		1.213***	0.474***	−0.006	−0.368***	−0.688***
Urban Ladino male		0.750***	0.296***	−0.011	−0.406***	−0.535***

*Note*: For the UVG samples, mean difference between ML and H, across four categories of H. For the USAC samples, mean difference between SH and LL, across four (rural Maya) or five (urban Ladino) categories of LL. Significance from the paired *t* test is indicated.

*P ≤ 0.05, ***P ≤ 0.001.

**Table 2:** Mean difference between sitting height (SH) and leg length (LL) within categories of education of the mother (USAC rural Maya children)

	Mean difference (SH − LL)		
	No education	Primary first to third	Primary fourth to sixth
Rural Maya female	1.399***	1.038***	1.070***
Rural Maya male	0.741***	0.755***	0.548***

*Note*: Mean difference between SH and LL, across the three categories of education of the mother. Significance from the paired *t* test is indicated.

***P ≤ .001.

**Table 3:** Mean difference in relative metatarsal length (Metatarsal length/Height) between categories of height (*H*) for the UVG samples

	Mean difference		
	H(−3/−2) − H(<−3)	H(−2/−1) − H(−3/−2)	H(>−1) − H(−2/−1)
Semi‐urban Maya female	−0.00616*	−0.00052	0.00143
Semi‐urban Maya male	−0.00204	0.00308	0.00093
Urban Ladino female	0.00388	0.00233	0.00038
Urban Ladino male	−0.00145	0.00018	0.00341

*Note*: No statistically significant differences were observed in the pairwise comparisons of the ANOVA test (Games‐Howell test).

*P ≤ .05.

**Table 4:** Sexual differences for sitting height (SH), leg length (LL) and height (H) across categories of maternal education

	Mean difference (female value − male value)					
ML	H(<−3)	H(−3/−2)	H(−2/−1)	H(>−1)		
UVG Semi‐urban Maya	0.332**	0.358***	0.230**	0.116		
UVG Urban Ladino	0.672***	0.315***	0.423***	0.134		
SH	LL(<−3)	LL(−3/−2)	LL(−2/−1)	LL(>−1)		
USAC Rural Maya	0.609**	0.465***	0.255*	0.323		
SH		LL(−3/−2)	LL(−2/−1)	LL(−1/0)	LL(0/1)	LL(>1)
USAC Urban Ladino		0.328	0.171	−0.027	0.074	−0.186

*Note*: Mean difference between sexes (male − female) for ML (UVG samples), and SH (USAC samples), across categories of H and LL, respectively. Significance from the independent samples *t* test is included. Positive values indicate higher female values.

*P ≤ .05, **P ≤ .01, ***P ≤ .001.

**Table 5:** Sexual differences for metatarsal length (ML) and sitting height (SH) across categories of height (H) and leg length (LL)

	Mean difference (female value − male value)		
	No education	Primary first to third	Primary fourth to sixth
SH	0.309**	−0.004	0.653*
LL	−0.343***	−0.288	0.131
H	−0.070	−0.210	0.364

*Note*: Mean difference between sexes (female − male) for the USAC rural Maya sample. Significance from the independent samples *t* test is included. Positive values indicate higher female values.

*P ≤ .05, **P ≤ .01, ***P ≤ .001.

## 4 DISCUSSION

Our main objective was to investigate the within population variation in the length of body segments, and thus, an important concern for the reliability of the results was if the UVG and USAC samples could be considered homogeneous. While the USAC urban and rural samples were chronologically homogeneous, data for the UVG study were taken in a 36 year period (1965‐1999), and its homogeneity could be compromised by temporal changes in the living conditions of the Guatemalan population during those years. A linear regression analysis was undertaken to evaluate the possible change through time of H and ML in the UVG subsamples, with decimal date of birth as the independent variable (Supporting Information 2). Statistical significance was observed for semi‐urban Maya children for H and ML, although a very low percentage of the variance in both variables was explained by date of birth (0%‐1.3%) (Supporting Table 2). Similar results have been recently obtained by Mansukoski et al. (2019), who studied all the available anthropometric records from the UVG Longitudinal Study. We consider that the changes in height and metacarpal length in the present samples of children (Supporting Figures 2 and 3), are small enough to allow considering these UVG samples as homogenous for the purpose of the present work, the study of the intra‐population variation between H and ML. These findings would be in agreement with the available anthropometric evidence that indicates absent or moderate temporal change in height for most of the Guatemalan population during the second half of the twentieth century and the first decade of the 21st century (Ríos, 2009; Ríos & Bogin, 2010) (Supporting Information 2). The homogeneity in SES was supported by the type of school (national schools with no fee for the low SES urban and semi‐urban UVG samples and for the USAC rural sample, vs private schools for the USAC urban sample), and by education of the mother for the USAC samples (see Section 2). The rural vs urban setting, as well as the Maya vs Ladino ascription are strongly associated to different levels of poverty within Guatemala, as reviewed in detail by Ríos (2009) and Ríos and Bogin (2010). For instance, the 1995 National Survey on Maternal and Child Health showed that the rate of stunting in preschool children changed from 35.3% (urban) to 56.6% (rural), and from 36.7% (Ladino) to 67.8% (Maya), for a total of 49.7% of all children, with similar differences present in previous and posterior surveys (Ríos & Bogin, 2010). Thus, the rural or urban setting and the Maya or Ladino ascription offer an important, general, SES indicator within a very unequal country like Guatemala. The contrast in SES and anthropometry between the UVG urban Ladino and the USAC urban Ladino children, beyond attending national schools or private schools, would be in agreement with the growing literature exploring inequality within large capital cities, with differences that can be similar to the rural vs urban contrast (Varea et al., 2018). With regard to the geographic variation in sample distribution, all the municipalities were located within one degree latitude, and within an altitude interval of approximately 1900 m, from 243 to 2128 m (within an interval of 967 m, from 1161 to 2128 m if we exclude one of the municipalities), with an average altitude for the eight municipalities of 1618 m (Supporting Information 1).

Another important factor that could affect the homogeneity of the subsamples studied was their age composition and the effect of puberty. Limb proportions change during growth but it seem to become established and stable between 10 and 12 years of age (Bogin & Rios, 2003). With regard to the age distribution, the subsamples compared did not present clear differences (Supporting Information 3). With regard to the influence of puberty, we re‐analyzed all the comparisons considering prepubescent and postpubescent children, and the results did not differ in comparison with those obtained with the total samples in statistical significance and in the pattern of change of the mean difference (Supporting Information 3). All these considerations supported the homogeneity of the subsamples and their validity to study the within population variation in the length of body segments.

The higher values of ML vs H in all comparisons, and of SH vs LL in most comparisons (Table 1, Figures 2, 3, 4, 5), indicate that the growth of ML and SH are prioritized over the growth of H and LL, respectively. The consistent pattern of increase in the mean difference of ML vs H and of SH vs LL with decreasing H and LL, further support the preservation of the growth of the ML and SH segments (Table 1, Figures 2, 3, 4, 5). The lack of change in relative ML length across the range of variation of H, except for one comparison, also indicates the preservation of the proportionality of the metatarsal segment relative to size. Our results for ML vs H are in agreement with previous studies in Andean and Himalayan populations, where it was observed that hand and foot lengths were conserved at the expense of forearm and lower leg lengths (Payne, Kumar, et al., 2018; Pomeroy et al., 2012). In the Himalayan study the preservation of proportionality of the hand segment relative to size was also observed (Payne, Kumar, et al., 2018).

With regard to SH vs LL, leg length has been the most studied body segment in growth and epidemiology research (Gunnell et al., 2003; Whitley, Martin, Smith, Holly, & Gunnell, 2012). In a previous study of Maya refugees that migrated to the US, a rapid increase of stature due primarily to an increase in relative LL was observed (Bogin et al., 2002). Here, we observe a differential response of the growth of SH and LL across the range of variation of LL. Interestingly, for the high SES urban Ladino children, a pattern of increase of the values of LL over SH was observed when LL was greater than −1 *z* score (Table 1 and Figure 5). The pattern of change in SH vs LL in rural Maya and urban Ladino children indicates the higher plasticity of LL in comparison with SH (Figures 4 and 5). In a previous study of highland and lowland Peruvian children it was also observed that SH was the less affected body segment in the highland children (Pomeroy et al., 2012). We also consider education of the mother, an important variable in biosocial health research, associated with child development measures and physical growth (Cauich‐Viñas et al., 2019; Fernald, Kariger, Hidrobo, & Gertler, 2012). For USAC rural Maya children, a decrease in SH and LL for males and females with decreasing education of the mother was observed, although the differences were statistically significant only for some comparisons and mostly for females (Supporting Information 4). The grouping of children according to categories of maternal education also supports the prioritization of the growth of SH over LL, with differences statistically significant in all cases (Table 2 and Figure 6), although a clear gradient of increase in the mean difference between both variables (Figures 2, 3, 4, 5) was not observed. An unbalanced sample size across the categories of education of the mother, with no representation of higher categories, may have influenced these results.

The change in the proportions of body segments is affected by several factors at play in migrant or temporal change studies (important differences in the overall environment between populations, genetic composition), and in high altitude studies (hypoxia, temperature). In particular, the variation in body size and limb proportions in relation to climate adaptation has been an important focus of research (Ruff, 1994), both in skeletal and living samples. For instance, the skeletal record shows a pattern of climate adaptation for hand and foot (Betti, Lycett, von Cramon‐Taubadel, & Pearson, 2015), with cold environments associated with shorter and broader hand elements. Recent studies also indicate the significance of hand and digit size and proportions for heat loss and dexterity during cold exposure, providing support for thermoregulation as an important factor contributing to variation in human hand proportions (Payne, Macintosh, & Stock, 2018a, 2018b). Other studies have supported differences in the socioeconomic environment for the explanation of changes in limb proportions in skeletal samples from different chronology (Jantz & Meadows Jantz, 2017; Macintosh, Pinhasi, & Stock, 2016), while other studies have supported the thrifty phenotype hypothesis over the predictions of the distal blood flow and cold adaptation models in the growth of different body segments in living populations (Payne, Kumar, et al., 2018; Pomeroy et al., 2012). The current work adds to this previous research by studying the growth of body segments within populations according to two general measures of overall living conditions, height‐ or leg length‐for‐age (H and LL), and maternal education. Our results could also be interpreted as the normal within population variation in the growth of ML vs H and SH vs LL (Figures 2, 3, 4, 5), reflecting a general allometry of the growth of body segments in all populations. In this regard, the moderate variation associated to maternal education (Figure 6), and the results from previous studies (Bogin et al., 2002; Payne, Kumar, et al., 2018; Pomeroy et al., 2012), support the thrifty phenotype hypothesis (Hales & Barker, 1992; Wells, 2013). According to this hypothesis, the growth of the head‐trunk segment would be prioritized due to the important organs that it houses (brain, heart, lungs), and the growth of the hand would be prioritized due to its manipulative function in human tool technology, thus offering an ultimate evolutionary explanation for the differential plasticity in the growth of body segments under different conditions of stress.

With regard to sexual differences, it has been suggested that sexual dimorphism in height is a proxy for environmental quality, an hypothesis based on the assumption of greater male sensitivity to suboptimal environment during growth and development, expressed as growth retardation (Nikitovic & Bogin, 2014; Stinson, 1985). This hypothesis has been tested previously in the UVG samples (Nikitovic & Bogin, 2014), where it was observed that a decreasing SES reduced or even eliminated statistically significant sexual dimorphism in height. In the present study, when values of H and LL were under −1 *z* scores, females presented higher ML and SH values than males, with most differences statistically significant (Table 4). Lack of statistically significant differences were observed for the category −1 to 0 *z* scores of H and LL, and higher male values were only observed in the high SES USAC urban Ladino sample for the highest categories of LL. When using maternal education as the grouping variable, for the category of no education, females present higher values of SH and lower values of LL than males (in both cases statistically significant differences, Table 5). These results could indicate greater male sensitivity for the growth of ML and SH, and for females could suggest a preservation of SH at the expense of LL.

In summary, the current study shows the differential preservation of metatarsal length and sitting height over height and leg length, respectively, in response to two general measures of the quality of the environment (height‐ or leg length‐for‐age and maternal education), within different samples of children. These findings add to previous studies in other populations (Payne, Kumar, et al., 2018; Pomeroy et al., 2012), and support the thrifty phenotype hypothesis, where the growth of some body segments would be prioritized due to their greater functional significance.

## AUTHOR CONTRIBUTIONS

L.R. and B.B. designed the study and directed implementation and data collection. L.R. and B.B. analyzed the data. L.R. drafted the manuscript. L.R., J.M.T., C.V., B.B. edited the manuscript for intellectual content and discussed the results.

## Supporting information


**Data S1**: Supporting Information

**Data S2**: Supporting Information
