# Supplementary material for: Plasticity in the growth of body segments in relation to height‐for‐age and maternal education in Guatemala
Source: Am J Hum Biol. 2019 Dec 19;32(4):e23376. doi: 10.1002/ajhb.23376 (PMC7507214; doi:10.1002/ajhb.23376)
Supplement: Supplementary file 1 — Data S1: Supporting Information [file AJHB-32-e23376-s001.docx]

**Supporting Information 1**

The location of the municipal seats of the six municipalities where children from the USAC study were measured (blue circles), and the location of San Pedro Sacatepequez and Guatemala City where children from the UVG study were measured (green circles, with black border for Guatemala City, where children from the USAC study were also measured), are shown in Supporting Figure 1. Data for latitude and longitude were plotted in a map of Guatemala in R with the packages “maps” and “mapdata”. The map [https://commons.wikimedia.org/-wiki/File:Guatemala_location_map.svg](https://commons.wikimedia.org/wiki/File:Guatemala_location_map.svg) was used to plot the borders of the departments. Data are summarized in Supporting Table 1.

**Supporting Figure 1**. Blue circles, from west to east: Cuilco, Retalhuleu, San Juan La Laguna, Sololá, San Juan Comalapa, San Bartolomé Milpas Altas. Green circles, from west to east: San Pedro Sacatepequez, Guatemala City.

**Supporting Table 1**. Geographical data for the municipal seats.

| **Municipality** | **Longitude†** | **Latitude†** | **Altitude, meters‡** |
| --- | --- | --- | --- |
| **Comalapa** | -90.8833298 | 14.7333304 | 2114 |
| **Cuilco** | -91.9666628 | 15.3999984 | 1161 |
| **San Bartolomé Milpas Altas** | -90.68 | 14.60611 | 2101 |
| **San Juan La Laguna** | -91.28333 | 14.7 | 1606 |
| **Sololá** | -91.1833326 | 14.7666636 | 2128 |
| **Retalhuleu** | -91.67778 | 14.53611 | 243 |
| **Guatemala** | -90.522713 | 14.628434 | 1501 |
| **San Pedro Sacatepequez** | -90.6442 | 14.6842 | 2097 |

†: decimal degrees, data obtained from <https://latitude.to/>. ‡: data obtained from <https://es-gt.topographic-map.com/>.

**Supporting Information 2**

The regression analysis for the UVG samples with decimal date of birth as the independent variable and the z-scores of height and metacarpal length as the dependent variables is summarized in Supporting Table 2. For all comparisons, a very low percentage of the variance in height and metacarpal length was explained by date of birth (from 0% to 1.3%), and significant regression equations were found only for semi-urban Maya children.

**Supporting Table 2**. Summary of the linear regression analyses, UVG subsamples.

| **Height** | **R Square** | **Unstandardized coefficient** | **P-value** |
| --- | --- | --- | --- |
| **Semi-urban Maya female** | 0.010 | 0.015 | 0.031* |
| **Semi-urban Maya male** | 0.013 | 0.014 | 0.005** |
| **Urban Ladino female** | 0.011 | 0.015 | 0.052 |
| **Urban Ladino male** | 0.004 | 0.009 | 0.204 |
| **Metatarsal length** |  |  |  |
| **Semi-urban Maya** | 0.010 | 0.014 | 0.032* |
| **Semi-urban Maya** | 0.011 | 0.014 | 0.010* |
| **Urban Ladino** | 0.000 | 0.001 | 0.889 |
| **Urban Ladino** | 0.000 | 0.003 | 0.688 |

*P ≤ 0.05, **P ≤ 0.01.

**Supporting Figure 2**. Bivariate plots and linear regression for z-scores of height and date of birth for the four subsamples.

We consider that the changes in height and metacarpal length (Supporting Figures 2 and 3), although statistically significant for semi-urban Maya children, are small enough to allow considering these UVG samples as homogeneous for the purpose of the present work, the study of intrapopulation variation between height and metacarpal length. These results would be in agreement with the recent findings by Mansukoski et al. (in press), who analyzed all the available measurements from the UVG Longitudinal Study (40,484 children) with SITAR (Cole, Donaldson & Ben-Shlomo, 2010), and observed a decrease in the height difference between the lowest (semi-urban Maya children) and highest (not analysed in the present study) SES groups. We observed a smaller temporal change in our subsample of semi-urban Maya children, which could be due to **Supporting Figure 3.** . Bivariate plots and linear regression for z-scores of metacarpal length and date of birth for the four subsamples.

differences in sampling between both studies. We only selected those children with both anthropometry and hand radiograph while Mansukoski et al. (in press) studied all the children with anthropometry, and we studied 6 to 15 years old children while Mansukoski et al. (in press) studied 3 to 19 years old children. A modest temporal change in height for some of the UVG subsamples would be in agreement with data summarized by Ríos (2009) and Ríos and Bogin (2010). For instance, data from the National Maternal and Child Health Survey (NMCHS) and Censo Nacional de Talla (CNT), shown in Supporting Tables 3 and 4, indicate high and similar levels of children under -2 z-scores of height between the years 1986-2002, with a clear decrease in comparison with previous years only in the 2015 surveys, providing a general overview of the living conditions in Guatemala during the last 30 years. The presence of a temporal change in height in some of the UVG samples could be related to the better living conditions of the Guatemala City and Guatemala Department population in comparison with population from other Departments (PNUD, 1998).

**Supporting Table 3**. Data from NMCHS (1987, 1995, 1999, 2002, 2009, 2015) (https://www.ine.gob.gt/images/2017/encuestas/ensmi2014_2015.pdf)

| Children under 5 years of age | 1987 | 1995 | 1998/99 | 2002 | 2008/2009 | 2014/15 |
| --- | --- | --- | --- | --- | --- | --- |
| Percentage < 2 z-scores height | 57.9* | 55.2 | 46.4* | 54.3 | 49.8 | 46.5 |

**Supporting Table 4**. Data from CNT (2015) (http://www.sesan.gob.gt/wordpress/informacion/descargas/iv-censo-nacional-de-talla-2015/)

| Children 6 to 10 years of age | 1986 | 2001 | 2008 | 2015 |
| --- | --- | --- | --- | --- |
| Percentage < 2 z-scores height | 51.1 | 49.7 | 45.6 | 37.6 |

**Supporting Information 3**

Limb proportions change during growth but adult proportions become established and stable by about 10 years and certainly by about 12 years (Bogin and Ríos, 2003). Two aspects of the effect of age on limb proportions that could impact the results presented were studied.

First, the age distribution of the subsamples compared are shown in Supporting Figures 4 and 5. It can be observed that, for each subsample (UVG semi-urban Maya females, UVG semi-urban Maya males, etc.), the z-score categories of height (UVG) and leg length (USAC) include children from all age groups in similar proportions, thus excluding the impact of the age distribution on the results presented in Figures 2-5 from the main text.

Second, the potential impact of puberty was evaluated. The reference sample from the First Zurich Longitudinal Study presented an age of peak height velocity (PHV) for girls and boys of 11.75 and 13.75 years respectively (Prader et al., 1988). Since the onset of puberty happens on average two years before the age at PHV (Bogin, 1999), the age of onset of puberty of children from the reference sample would be approximately 9.75 and 11.75 years for girls and boys. For the high SES USAC urban Ladino children, these ages would approximate their age of onset of puberty, so that we could divide this sample in pre- and post- pubescent. For the Guatemalan low SES samples (UVG semi-urban Maya and urban Ladino, USAC rural Maya), the use of these ages as upper limits would result in prepubescent samples, especially since previous studies on the UVG samples have shown that the low SES samples present a later onset of puberty (Bogin et al., 1989, 1992). By using the same ages as lower limits for the same low SES Guatemalan samples, there would be an admixture

**Supporting Figure 4.** Age distribution in percentages of the four height categories compared within each of the four UVG subsamples.

**Supporting Figure 5.** Age distribution in percentages of the four (rural Maya) or five (urban Ladino) leg length categories compared within each of the four USAC subsamples.

of mainly postpubescent but also prepubescent and pubescent children, but for sake of simplicity we consider these samples as postpubescent. As shown in Supporting Tables 5-7, the re-analyses of these prepubescent and postpubescent samples presented the same patterns that those obtained with the total samples. This is illustrated with two examples in Supporting Figures 6 and 7. These results supported the lack of influence of puberty on the results presented in Figures 2-5 from the main text.

**Supporting Table 5**. Mean difference and significance of the paired t-test for the UVG total, prepubescent and postpubescent samples.

|  | **Sample** | **H (< -3)** | **H (-3 / -2)** | **H (-2 / -1)** | **H (> -1)** |
| --- | --- | --- | --- | --- | --- |
| **Semi-urban Maya female** | Total | 1.277*** | 1.065*** | 0.716*** | 0.402*** |
|  | Prepubescent | 1.402*** | 1.186*** | 0.790*** | 0.507*** |
|  | Postpubescent | 1.193*** | 0.990*** | 0.650*** | 0.318** |
| **Semi-urban Maya male** | Total | 0.859*** | 0.601*** | 0.476*** | 0.230*** |
|  | Prepubescent | 0.948*** | 0.680*** | 0.552*** | 0.188* |
|  | Postpubescent | 0.553** | 0.461*** | 0.340*** | 0.265* |
| **Urban Ladino female** | Total | 1.725*** | 1.117*** | 0.871*** | 0.426*** |
|  | Prepubescent | 1.834*** | 1.162*** | 0.979*** | 0.495*** |
|  | Postpubescent | 1.626*** | 1.033*** | 0.794*** | 0.312** |
| **Urban Ladino male** | Total | 0.993*** | 0.788*** | 0.508*** | 0.355*** |
|  | Prepubescent | 1.089*** | 0.855*** | 0.493*** | 0.371*** |
|  | Postpubescent | 0.535 | 0.558*** | 0.562*** | 0.415*** |

*P ≤ 0.05, **P ≤ 0.01, ***P ≤ 0.001.

**Supporting Table 6**. Mean difference and significance of the paired t-test for the USAC rural Maya total, prepubescent and postpubescent samples.

|  | **Sample** | **LL (< -3)** | **LL (-3 / -2)** | **LL (-2 / -1)** | **LL (> -1)** |
| --- | --- | --- | --- | --- | --- |
| **Rural Maya female** | Total | 1.825*** | 1.429*** | 0.920*** | 0.619*** |
|  | Prepubescent | 1.684*** | 1.401*** | 0.884*** | 0.354* |
|  | Postpubescent | 1.940*** | 1.453*** | 0.984*** | 1.271** |
| **Rural Maya male** | Total | 1.398*** | 0.992*** | 0.576*** | -0.084 |
|  | Prepubescent | 1.270*** | 0.926*** | 0.541*** | -0.201 |
|  | Postpubescent | 1.618*** | 1.164*** | 0.753*** | 0.387 |

*P ≤ 0.05, **P ≤ 0.01, ***P ≤ 0.001.

**Supporting Table 7.** Mean difference and significance of the paired t-test for the USAC urban Ladino total, prepubescent and postpubescent samples.

|  | **Sample** | **LL (-3 / -2)** | **LL (-2 / -1)** | **LL (-1 / 0)** | **LL (0 / 1)** | **LL (> 1)** |
| --- | --- | --- | --- | --- | --- | --- |
| **Urban Ladino female** | Total | 1.257*** | 0.458*** | -0.211*** | -0.395*** | -0.702*** |
|  | Prepubescent | -0.374 | 0.099 | -0.228* | -0.594*** | -0.809*** |
|  | Postpubescent | 1.427*** | 0.580*** | 0.110 | -0.274** | -0.607*** |
| **Urban Ladino male** | Total | 0.714*** | 0.345*** | -0.024 | -0.406*** | -0.596*** |
|  | Prepubescent | 0.535** | 0.181* | -0.166** | -0.447*** | -0.814*** |
|  | Postpubescent | 1.094* | 0.549*** | 0.182* | -0.339** | -0.348 |

*P ≤ 0.05, **P ≤ 0.01, ***P ≤ 0.001.

**Supporting Information 4**

In Supporting Table 8 we show the mean difference and significance from the pairwise comparisons from ANOVA for SH and LL between categories of education of the mother. The mean difference between the highest and lowest maternal education categories is statistically significant for SH and LL for females, and for LL for males. The other pairwise comparisons show mixed results, with significant differences only for females.

**Supporting Table 8. Mean difference for SH and LL between categories of education of the mother (USAC rural Maya children)**. Mean differences and significances from the pairwise comparisons from ANOVA are shown.

| **Mean difference** | **Female SH** | **Female LL** | **Male SH** | **Male LL** |
| --- | --- | --- | --- | --- |
| **(Primary 4^th-6th^) – (No education)** | 0.651* | 0.864* | 0.308 | 0.529* |
| **(Primary 4^th-6th^) – (Primary 1^st^-3^rd^)** | 0.766** | 0.645* | 0.108 | 0.323 |
| **(Primary 1^st^-3^rd^) – (No education)** | -0.115 | 0.218 | 0.199 | 0.205 |

*P ≤ 0.05, **P ≤ 0.01.

**Supporting Figure 6.** Comparison of z-scores between metatarsal length (ML) and height (H) across four categories of H (<-3, -3/-2, -2/-1, >-1) for UVG semi-urban Maya females: total sample (top row), prepubescent sample (middle row), and postpubescent sample (bottom row). See Figure 1 (main text) for a description of the details of the plots, and Supporting Table 5 for significance of the paired t-tests.

**Supporting Figure 7.** Comparison of z-scores between sitting height (SH) and leg length (LL) across four categories of LL (<-3, -3/-2, -2/-1, >-1) for USAC rural Maya males: total sample (top row), prepubescent sample (middle row), and postpubescent sample (bottom row). See Figure 1 (main text) for a description of the details of the plots, and Supporting Table 6 for significance of the paired t-tests.

**References**

Bogin, B. (1999). *Patterns of human growth, 2^nd^ edition.* Cambridge Studies in Biological Anthropology, Vol. 23. Cambridge University Press.

Bogin, B., Sullivan, T., Hauspie, R., & Macvean, R. B. (1989). Longitudinal growth in height, weight, and bone age of Guatemalan Ladino and Indian schoolchildren. *American Journal of Human Biology*, *1*(1), 103-113.

Bogin, B., Wall, M., & MacVean, R. B. (1992). Longitudinal analysis of adolescent growth of Ladino and Mayan school children in Guatemala: effects of environment and sex. *American Journal of Physical Anthropology*, *89*(4), 447-457.

Bogin, B., & Rios, L. (2003). Rapid morphological change in living humans: implications for modern human origins. *Comparative Biochemistry and Physiology a-Molecular & Integrative Physiology, 136*(1), 71-84. doi: 10.1016/s1095-6433(02)00294-5

Cole, T. J., Donaldson, M. D., & Ben-Shlomo, Y. (2010). SITAR—a useful instrument for growth curve analysis. *International Journal of Epidemiology*, 39(6), 1558-1566.

Mansukoski, L., Johnson, W., Brooke-Wavell, K., Galvez-Sobral, J.A., Furlán, L., Cole, T.J. & Bogin, B. (in press). Four decades of socioeconomic inequality and secular change in the physical growth of Guatemalans. *Public Health Nutrition*.

PNUD, Programa Naciones Unidas para el Desarrollo. 1998. Guatemala: Los contrastes del desarrollo humano. United Nations.

Prader, A., Largo, R. H., Molinari, L., & Issler, C. (1989). Physical growth of Swiss children from birth to 20 years of age. First Zurich longitudinal study of growth and development. *Helvetica paediatrica acta. Supplementum, 52*, 1-125.

Ríos, L. (2009). Guatemala: una revisión de las fuentes antropométricas disponibles. *Historia Agraria*, 47, 217-238.

Ríos, L., & Bogin, B. (2010). An anthropometric perspective on Guatemalan ancient and modern history. *Living standards in Latin American history: height, welfare, and development, 1750–2000*, 273-309.
