# Supplementary material for: Plasticity in the growth of body segments in relation to height‐for‐age and maternal education in Guatemala
Source: Am J Hum Biol. 2019 Dec 19;32(4):e23376. doi: 10.1002/ajhb.23376 (PMC7507214; doi:10.1002/ajhb.23376)
Supplement: Supplementary file 2 — Data S2: Supporting Information [file AJHB-32-e23376-s002.docx]

**Table 1. Descriptive statistics for the UVG samples.** ML: metatarsal length. H: height.

| UVG semi-urban Maya |  |  |  |  |  |  |  |  |  |  |
| --- | --- | --- | --- | --- | --- | --- | --- | --- | --- | --- |
|  | Female |  |  |  |  | Male |  |  |  |  |
|  | N | ML Mean | SD | H Mean | SD | N | ML Mean | SD | H Mean | SD |
| 6 | 12 | -0.70424 | 1.13968 | -1.48083 | 1.25864 | 14 | -1.16716 | 1.00436 | -1.43655 | 0.92881 |
| 7 | 87 | -0.76394 | 0.90908 | -1.64820 | 1.03198 | 98 | -0.98512 | 1.05793 | -1.65592 | 0.97187 |
| 8 | 86 | -1.20265 | 0.88701 | -2.26577 | 1.00839 | 108 | -1.24470 | 0.89852 | -1.81794 | 0.95330 |
| 9 | 65 | -1.47859 | 0.81091 | -2.49415 | 0.87344 | 59 | -1.62545 | 0.96761 | -2.28096 | 0.77621 |
| 10 | 54 | -0.84925 | 1.02063 | -1.61275 | 1.05994 | 60 | -1.63540 | 0.91738 | -2.11378 | 0.91848 |
| 11 | 38 | -1.02675 | 1.05744 | -1.82891 | 1.10638 | 45 | -1.45924 | 1.12892 | -2.10976 | 1.13777 |
| 12 | 51 | -1.23691 | 1.00662 | -1.80665 | 1.08948 | 54 | -1.60271 | 0.95780 | -1.87146 | 0.94294 |
| 13 | 39 | -1.17144 | 0.85140 | -1.90831 | 0.88614 | 60 | -1.03301 | 1.06746 | -1.49493 | 0.93615 |
| 14 | 22 | -1.54616 | 1.13089 | -2.67388 | 0.85162 | 41 | -1.01735 | 0.92444 | -1.50842 | 0.85919 |
| 15 | 16 | -2.07634 | 0.84445 | -2.73196 | 0.92380 | 46 | -1.48554 | 0.77754 | -1.87938 | 0.59133 |
| UVG urban Ladino |  |  |  |  |  |  |  |  |  |  |
|  | Female |  |  |  |  | Male |  |  |  |  |
|  | N | ML Mean | SD | H Mean | SD | N | ML Mean | SD | H Mean | SD |
| 6 | 23 | -0.47029 | 0.97988 | -1.05685 | 1.25089 | 19 | -0.96548 | 0.67833 | -1.45051 | 1.10318 |
| 7 | 56 | -0.53569 | 0.96285 | -1.50351 | 1.00219 | 68 | -0.73964 | 1.23294 | -1.37305 | 1.21013 |
| 8 | 56 | -0.63032 | 1.01462 | -1.76470 | 1.13458 | 82 | -0.92961 | 0.99800 | -1.62996 | 1.01953 |
| 9 | 46 | -0.93195 | 0.97215 | -2.01261 | 1.27076 | 52 | -1.13670 | 1.11412 | -1.81028 | 1.02964 |
| 10 | 36 | -0.76412 | 1.00437 | -1.45351 | 1.18210 | 42 | -1.48911 | 0.96440 | -1.91919 | 1.13321 |
| 11 | 35 | -0.59359 | 1.14320 | -1.59014 | 1.31910 | 39 | -1.13102 | 1.06704 | -1.68646 | 1.14997 |
| 12 | 38 | -0.81629 | 0.99151 | -1.37821 | 1.17802 | 54 | -0.92928 | 1.27075 | -1.35633 | 1.05539 |
| 13 | 16 | -0.85589 | 0.99142 | -1.72196 | 1.00294 | 20 | -0.52650 | 1.02978 | -1.03803 | 0.94684 |
| 14 | 12 | -0.79918 | 0.60819 | -1.80992 | 1.22338 | 12 | -1.51653 | 0.73230 | -2.08023 | 0.78240 |
| 15 | 7 | -1.37796 | 0.94238 | -2.11626 | 1.04157 | 9 | -1.45482 | 0.69450 | -2.16737 | 0.73092 |

**Table 2. Descriptive statistics for the USAC samples.** SH: Sitting height. LL: leg length.

| USAC rural Maya |  |  |  |  |  |  |  |  |  |  |
| --- | --- | --- | --- | --- | --- | --- | --- | --- | --- | --- |
|  | Female |  |  |  |  | Male |  |  |  |  |
| Age | N | SH Mean | SD | LL Mean | SD | N | SH Mean | SD | LL Mean | SD |
| 6 | 52 | -0.68750 | 1.06173 | -1.81481 | 1.29309 | 44 | -0.98375 | 1.14671 | -2.00877 | 1.00077 |
| 7 | 51 | -0.92863 | 1.04786 | -2.18373 | 1.24239 | 52 | -1.03956 | 0.91613 | -1.83739 | 1.22105 |
| 8 | 50 | -1.23849 | 1.27606 | -2.08380 | 1.15918 | 38 | -1.14925 | 1.31912 | -1.47459 | 1.22390 |
| 9 | 53 | -0.89251 | 1.37124 | -2.06340 | 1.04775 | 50 | -1.52302 | 0.91757 | -2.08187 | 0.75399 |
| 10 | 42 | -1.14560 | 1.67858 | -2.27738 | 1.07136 | 50 | -1.30526 | 0.80230 | -1.87120 | 0.97480 |
| 11 | 38 | -0.75945 | 1.10024 | -2.17053 | 0.95679 | 42 | -1.30914 | 1.30302 | -1.74700 | 1.16148 |
| 12 | 28 | -0.58595 | 0.94432 | -2.46036 | 1.02232 | 42 | -1.42173 | 0.86797 | -2.20921 | 0.79128 |
| 13 | 30 | -0.68369 | 0.80558 | -2.69267 | 0.77517 | 32 | -1.08841 | 0.98086 | -1.95713 | 0.81870 |
| 14 | 19 | -1.44897 | 0.85459 | -2.96263 | 0.80795 | 17 | -0.86590 | 0.96702 | -2.27233 | 0.90814 |
| 15 | 15 | -1.83845 | 0.99687 | -2.92267 | 0.86304 | 11 | -1.15683 | 0.77127 | -2.76932 | 1.01857 |
| USAC urban Ladino**†** |  |  |  |  |  |  |  |  |  |  |
|  | Female |  |  |  |  | Male |  |  |  |  |
| Age | N | SH Mean | SD | LL Mean | SD | N | SH Mean | SD | LL Mean | SD |
| 6 | 41 | -0.19309 | 1.24888 | 0.10659 | 1.04235 | 32 | -.29219 | .973601 | -0.49342 | 1.13112 |
| 7 | 51 | -0.25412 | 1.14044 | 0.09980 | 1.20742 | 50 | -.43915 | 1.132865 | -0.37483 | 1.02469 |
| 8 | 81 | -0.60334 | 1.24370 | -0.17434 | 0.88273 | 91 | -.92292 | 1.050554 | -0.57377 | 0.86498 |
| 9 | 92 | -0.50379 | 1.18448 | -0.12047 | 1.01600 | 90 | -.56128 | 1.033101 | -0.32570 | 0.88813 |
| 10 | 88 | -0.21853 | 1.35761 | 0.09160 | 1.24845 | 78 | -.66296 | .913239 | -0.57759 | 0.89217 |
| 11 | 82 | 0.04122 | 1.29715 | -0.00980 | 1.01510 | 79 | -.46953 | .964053 | -0.37665 | 0.89227 |
| 12 | 81 | 0.28988 | 0.95825 | -0.01978 | 0.87619 | 68 | -.53584 | 1.018995 | -0.30342 | 1.05262 |
| 13 | 61 | -0.18344 | 0.86188 | -0.47506 | 1.08529 | 63 | -.09485 | 1.148074 | -0.28049 | 0.98563 |
| 14 | 69 | -0.53696 | 0.94500 | -0.97082 | 0.96921 | 40 | .02125 | .904468 | -0.34850 | 1.21889 |
| 15 | 82 | -0.69512 | 0.90929 | -1.00150 | 0.97001 | 69 | -.14359 | .898660 | -0.69623 | 0.98565 |

**†**The only eight children from the USAC urban Ladino sample with LL z-score values under -3 were not used in the analysis presented in the main text.
